# Oxidation of the major allergen Ses-i-2 from sesamum indicum: effect on the conformation, stability and dendritic cell stimulation activity

**DOI:** 10.1186/2045-7022-1-S1-O11

**Published:** 2011-08-12

**Authors:** Vincenzo De Filippis, Daniela Galla, Chiara D'Orlando, Paola Brun, Ignazio Castagliuolo

**Affiliations:** 1 University of Padova, Dept. of Pharmaceutical Sciences, Padova, Italy; 2 University of Padova, Dept. of Histology, Microbiology and Medical Biotechnologies, Padova, Italy

## 

Despite the medical and social impact of allergies to plant seed storage proteins, the molecular bases of these diseases are still largely unknown. Here we have purified to homogeneity large quantities (10-30 mg) of Ses-i-2, the major allergen from sesame seeds. Interestingly, Ses-i-2 contains an unusually high content of methionines (16%) and arginines (13%). The results of a thorough chemical, conformational and stability characterization indicate that Ses-i-2 possesses a highly helical secondary structure, cross-linked by five disulfide bridges, and that it is highly stable to both chemical and thermal denaturation. For instance, a melting temperature of 62°C could be measured only at pH 2.0 and in the presence of 6 M Gnd-HCl. The stability to denaturation is also paralleled by the extraordinary resistance of Ses-i-2 to proteolysis by digestive, blood coagulation, bacterial, lysosomal and leukocyte proteases. Furthermore, fluoresceinated Ses-i-2 is able to transverse intestinal epithelial cells. Finally, Ses-i-2 downregulates IL-12 production in cultured dendritic cells while increasing IL-10 and IL-4 levels, typical of a T helper type-2 response that is associated with the production of IgE antibodies.

To investigate a possible role of oxidation on Ses-i-2 structure and allergenicity, all Met-residues were selectively and quantitatively oxidised to methionine sulfoxide by treatement with 50 mM of H2O2 or with leukocyte mieloperoxidase-0.5 mM H2O2. Strikingly, the structure of oxidised Ses-i-2 (Ses-i-2-OX) is dramatically altered and Ses-i-2-OX is only marginally stable to chemical and thermal denaturants. Ses-i-2-OX also becomes immediately degraded by all proteases tested. Finally, Ses-i-2-OX has an opposite effect on cytokine production by dendritic cells, with a resulting T-helper type-1 response, usually associated with the production of IgG antibodies.

These findings suggest that individuals allergic to Ses-i-2 might have compromised oxidative machinery that allows Ses-i-2 to reach the intestinal immune system in its intact form, thus triggering the allergic response.

